# The association between bacteria and outcome and the influence of sampling method, in people with a diabetic foot infection

**DOI:** 10.1007/s15010-022-01884-x

**Published:** 2022-07-22

**Authors:** Meryl Cinzía Tila Tamara Gramberg, Shaya Krishnaa Normadevi Mahadew, Birgit Ilja Lissenberg-Witte, Marielle Petra Bleijenberg, Jara Rebekka de la Court, Jarne Marijn van Hattem, Louise Willy Elizabeth Sabelis, Rimke Sabine Lagrand, Vincent de Groot, Martin Den Heijer, Edgar Josephus Gerardus Peters

**Affiliations:** 1 Amsterdam UMC Location Vrije Universiteit Amsterdam, Infectious Diseases, De Boelelaan 1117, Amsterdam, The Netherlands; 2 Amsterdam Movement Sciences, Rehabilitation and Development, Amsterdam, The Netherlands; 3 Amsterdam Infection and Immunity, Infectious Diseases, Amsterdam, The Netherlands; 4 Amsterdam UMC Location Vrije Universiteit Amsterdam, Epidemiology and Data Science, De Boelelaan 1117, Amsterdam, The Netherlands; 5 Amsterdam UMC Location University of Amsterdam Medical Microbiology and Infection Prevention, Meibergdreef 9, Amsterdam, The Netherlands; 6 Amsterdam UMC Location Vrije Universiteit Amsterdam, Rehabilitation Medicine, De Boelelaan 1117, Amsterdam, The Netherlands; 7 Amsterdam UMC Location Vrije Universiteit Amsterdam, Endocrinology, De Boelelaan 1117, Amsterdam, The Netherlands; 8 Amsterdam UMC Center for Diabetic Foot Complications (ACDC), Amsterdam, The Netherlands

**Keywords:** Diabetes mellitus, Diabetic foot infection, Bacteria, Amputation, Mortality

## Abstract

**Purpose:**

Different bacteria lead to divers diabetic foot infections (DFIs), and some bacteria probably lead to higher amputation and mortality risks. We assessed mortality and amputation risk in relation to bacterial profiles in people DFI and investigated the role of sampling method.

**Methods:**

We included people (> 18 years) with DFI in this retrospective study (2011–2020) at a Dutch tertiary care hospital. We retrieved cultures according to best sampling method: (1) bone biopsy; (2) ulcer bed biopsy; and (3) swab. We aggregated data into a composite determinant, consisting of unrepeated bacteria of one episode of infection, clustered into 5 profiles: (1) *Streptococcus* and *Staphylococcus aureus*; (2) coagulase-negative *Staphylococcus*, *Cutibacterium*, *Corynebacterium* and *Enterococcus*; (3) gram-negative; (4) Anaerobic; and (5) less common gram-positive bacteria. We calculated Hazard Ratio’s (HR’s) using time-dependent-Cox regression for the analyses and investigated effect modification by sampling method.

**Results:**

We included 139 people, with 447 person-years follow-up and 459 episodes of infection. Sampling method modified the association between bacterial profiles and amputation for profile 2. HR’s (95% CI’s) for amputation for bacterial profiles 1–5: 0.7 (0.39–1.1); stratified analysis for profile 2: bone biopsy 0.84 (0.26–2.7), ulcer bed biopsy 0.89 (0.34–2.3), swab 5.9*(2.9–11.8); 1.3 (0.78–2.1); 1.6 (0.91–2.6); 1.6 (0.58–4.5). HR’s (95% CI’s) for mortality for bacterial profiles 1–5: 0.89 (0.49–1.6); 0.73 (0.38–1.4); 2.6*(1.4–4.8); 1.1(0.58–2.2); 0.80(0.19–3.3).

**Conclusions:**

In people with DFI, there was no association between bacterial profiles in ulcer bed and bone biopsies and amputation. Only in swab cultures, low-pathogenic bacteria (profile 2), were associated with a higher amputation risk. Infection with gram-negative bacteria was associated with a higher mortality risk. This study underlined the possible negative outcome of DFI treatment based on swabs cultures.

## Introduction

In people with diabetes, an ulcer with foot infection is the most prominent risk factor for lower extremity amputation [1–3]. Different bacterial species are thought to lead to clinically diverse infections with subsequent amputation risks [4–8]. In severe infections (including osteomyelitis), grade 4 of the International Working Group on the Diabetic Foot (IWGDF) diabetic foot infection classification, 77 to 90% of patients will undergo amputation [9–11]. Five-year mortality after (major) amputation is as high as 90% [12–15]. Insight in the association between different bacterial species and outcome is, therefore, of great importance, and might have implications for everyday practice.

In temperate climates, *Staphylococcus aureus* and (beta-haemolytic) *Streptococcus* are the most commonly identified pathogens in diabetic foot infections (DFIs) [6, 16]. Infections are usually acute, classified as mild to moderate and formation of pus is possible [5]. In daily practice, coagulase-negative *Staphylococcus* (CNS), *Corynebacterium*, *Enterococcus* and *Cutibacteria* (formerly *Propionibacterium*) are commonly present in ulcer cultures, but are usually considered colonizing, rather than pathogenic bacteria. However, when present in deep infections including osteomyelitis, they can be pathogens [6, 16–18]. Gram-negative bacteria cause approximately one third of DFIs, and anaerobes are found in a smaller minority of DFIs [4, 17, 19, 20]. Infections with these micro-organisms are usually chronic and more severe [21].

The presence of clinical and systemic signs of inflammation are used in the diagnosis of a skin and soft tissue DFI. Since all ulcers are colonized with bacteria, culture results of ulcers are used to guide antimicrobial therapy only, and cannot be used for diagnosis. Reference standard for osteomyelitis is the presence of signs of inflammation combined with a positive aseptically obtained percutaneous bone biopsy obtained through intact skin, adjacent the ulcer [22, 23]. In case of osteomyelitis, such a bone biopsy is preferred, and ulcer bed biopsy is considered second best. This is, however, based on limited data [23]. Whether bone culture directed antimicrobial therapy leads to better outcomes than ulcer bed culture directed therapy, is currently under investigation [24]. Ulcer bed biopsy is preferred in case of soft tissue infection without osteomyelitis [22, 23]. Swabs, although often used in cases with and without osteomyelitis, are considered inferior for detection of causative bacteria of DFI. Swabs are prone to culturing colonizing flora and causative bacteria could be missed [20, 25–28]. Proper identification of causative bacteria is essential to guide antibiotic therapy and to lower the risk of adverse outcomes. Therefore, sampling method (bone biopsy, ulcer bed biopsy or swab), is an important factor to consider, while studying the relation between bacterial profiles and amputation and mortality. In this retrospective study, we investigated associations between bacterial profiles and amputation and mortality risk, and we investigated the role of sampling method in this relation.

## Methods

We conducted a retrospective cohort study at the Amsterdam University Medical Centres location VUmc. We retrieved medical records from patients (≥ 18 years of age) who presented with a DFI between March the 16th 2011 and January 1st 2020 using the International Classification of Diseases codes 10th edition and the Dutch Diagnosis Treatment Code for ‘diabetes’ and ‘infection’. We included a person if DFI was diagnosed and DFI cultures were available. The diagnosis of infection was stated by an internist, rehabilitation physician or vascular surgeon and based on a combination of clinical findings of infection (swelling, pain, redness, warmth, purulent discharge, foul smell), increased systemic inflammatory biomarkers and/or abnormalities of the foot on imaging suggesting tissue and/or bone infection [23]. If no material for culture was taken or if culture results were missing, a participant was excluded. Participant follow-up started the date cultures were taken, and follow-up was until database closure (January 1st 2020) or until the participant deceased or was lost to follow up.

Most participants were treated by a multidisciplinary team consisting of an internist, rehabilitation physician, vascular surgeon, podiatrist, orthopaedic shoe technician and casting technician. Treatment consisted of antimicrobial therapy with or without surgery, in combination with appropriate offloading, vascular intervention if required and proper ulcer dressing.

### Demographics, sampling methods, cultures and episodes

We obtained participant characteristics (gender, age, type of diabetes, duration of diabetes and history of amputation) using electronic medical records. Sampling methods were: percutaneous bone biopsy, taken adjacent to the ulcer through intact disinfected skin or in the operating theatre, ulcer bed biopsy of debrided and rinsed ulcer beds, or swabs of debrided and rinsed ulcer beds. Material obtained by any of these methods was cultured, using standard techniques for culturing, identification of bacteria and to test antimicrobial sensitivity. These techniques included inoculating the sample on agar plates and in broth, followed by incubation in aerobic and anaerobic environment. We recorded sampling method (bone biopsy, ulcer bed biopsy, swab), if osteomyelitis as stated by the treating physician was present, and culture results. Similar bacterial species that were cultured at the same time in material from different sampling methods of the same location, were only counted in the best available sampling method. Best available sampling method according to the International Working Group on the Diabetic Foot (IWGDF) was bone biopsy in case of osteomyelitis, and ulcer bed biopsy if bone biopsy was not performed or if the participant had soft tissue infection without osteomyelitis [23]. We only used cultures from swabs if neither bone biopsy nor ulcer bed biopsy cultures were available.

**Fig. 1:**
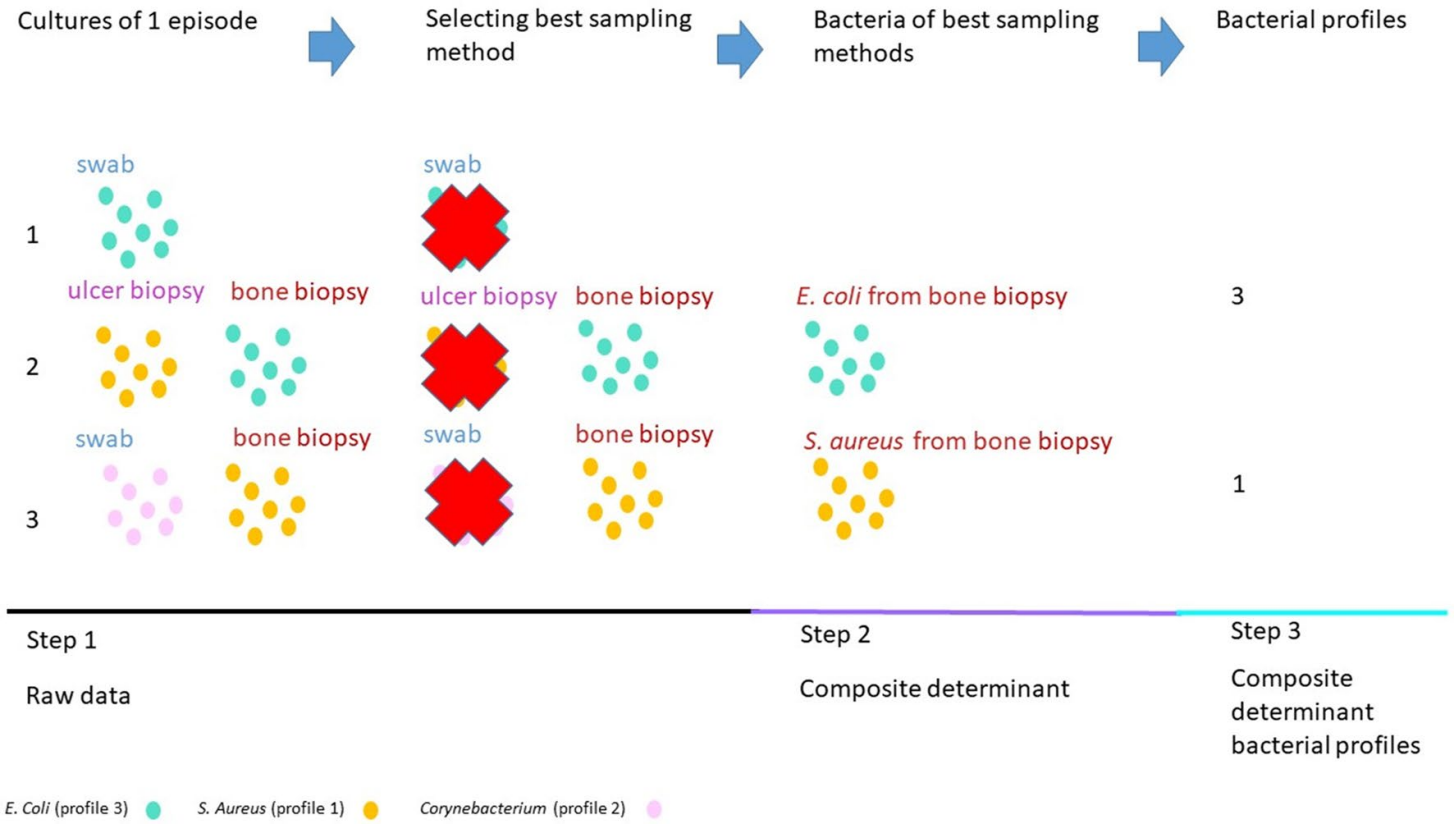
Step 1: cultures obtained from one participant during the course of 1 infection episode. There were 3 cultures obtained during this episode. 1. Swab: *E.coli*. 2. Ulcer bed: *S. aureus* and bone biopsy: *E. coli*. 3. Swab: *Corynebacterium* and bone biopsy: *S. aureus*. Step 2. Selecting best sampling method. Bone biopsy prevails over swab and ulcer bed biopsy, we therefore discarded the results of swab and ulcer bed biopsy (red crosses). Step 3. We created a composite determinant consisting of bacteria from best sampling method, and in which we removed duplicate bacterial species, e.g., if a *S. aureus* was cultured twice during one infective episode, we only counted this bacterial species ones (unrepeated bacterial species). After deduplication of bacterial species we grouped these bacteria into one of the five profiles. *S. aureus* causes acute, usually mild, infections and is grouped with Streptococcus in profile 1, and *E. coli* is a gram-negative bacterium, causing usually severe infection and is grouped in profile 3. For the analyses we used profile 1 and profile 3 in this example

We grouped cultures in episodes of infection. We defined one episode as all cultures from the same DFI within 3 month time. For the analysis of data we constructed a composite determinant. The composite determinant consisted of pooled unrepeated bacteria of one episode (Fig. 1).

### Outcomes

We recorded the number of episodes of infection for all participants during follow-up.

Primary outcomes were amputation and mortality. We used surgical procedure codes to identify patients who underwent lower extremity amputation during the study period, excluding amputation due to gangrene due to ischemia without infection, and we recorded level of amputation: minor (distal to the malleoli) or major (proximal to the malleoli) [29], and we gathered information regarding mortality from medical records.

We also recorded if a participant was lost to follow up. The last documentation date in the participant’s medical records, counted as date of censoring.

### Analyses

We analysed data using R version 4.0.3. To explore bacterial profiles, we grouped individual bacterial species into 5 profiles, according to perceived virulence and morphology. Profile 1: *Streptococcus and Staphylococcus,* infections are usually acute, and mild and the formation of pus is possible. Profile 2: Coagulase-negative *staphylococcus*, *Cutibacterium*, *Corynebacterium* and *Enterococcus*, these bacteria are usually considered low-pathogenic. Profile 3: gram-negative bacteria, usually these infections are considered more severe and polymicrobial. Profile 4: anaerobic bacteria, infections are usually considered severe. Profile 5: less common gram-positive bacteria (e.g., *Actinomyces, Lactococcus)* [5]. We collected culture results of one infective episode of one participant, than we selected only the results of the best available sampling method, and we aggregated these selected results into a composite determinant, and used this composite determinant for the analyses, i.e., if in one participant respective of one episode more than one profile was encountered in the best sampling method (e.g., *S. aureus* (profile 1) and *E. coli* (profile 3) were encountered both in bone biopsy), than both these profiles were used for the analyses. Aggregation of data into bacterial profiles and the composite determinant is shown in Fig. 1.

We calculated incidence rates for infection per profile per person year for profiles 1–5, and used time-dependent Cox regression to investigate the association between bacterial profiles (the exposure) and time to amputation and death (the outcomes). For this type of regression analyses we used the exact dates of culturing and the appearance of the outcome, in this way we included the time component in the analyses. Effect sizes were reported as Hazard Ratios (HRs) including 95% confidence intervals (95% CI’s). For each of the bacterial profiles we created a dichotomous variable and scored them as being present in the culture or not. We investigated effect modification for the variables: best sampling method and the presence of osteomyelitis. As a sensitivity analysis, we investigated the relation between bacterial profiles and amputation and mortality in participants without a history of amputation.

## Results

### Baseline characteristics and cultures

We screened records of 298 people with suitable International Classification of Disease-10 and Diagnosis Treatment Codes, of which 202 had a registered foot ulcer. Of these people, we could obtain microbiological data of 139 people, and included them in the study. Baseline demographics are displayed in Tables 1.  Table 2 shows culture results, and bacterial profile frequencies in positive cultures.

**Table 1:** Baseline characteristics

Baseline characteristics	*N*, (%)—unless otherwise specified
Male gender	95 (68.3)^a^
Age in years, mean (SD)	71.3 (11.8)
Type 2 diabetes	126 (90.6)^a^
Duration of diabetes in years, median (interquartile range)^b^	18 (8–29)
History of amputation	38 (27.3)^a^

^a^Percentage of the total population of 139 people

^b^Duration of diabetes calculated from the study start date

**Table 2:** Cultures, episodes, follow-up and outcomes

Cultures	
Total amount of cultures taken during study	3310
Positive cultures	2238 (67.6)^a^
Polymicrobial	1554 (69.4)^b^
Cultures of best sampling method after deduplication	1408
Frequencies of bacterial profiles in cultures	
Profile	Frequency
1	478 (20.5)^b^
2	248 (11.1)^b^
3	473 (21.1)^b^
4	168 (7.5)^b^
5	41 (1.8)^b^
Episodes of infection	
Total episodes	459
Median episodes per participant (interquartile range)	2 (1–4)
Episodes of infection with osteomyelitis	155 (33.8)^c^
Follow-up	
Total follow-up, person-years	447
Lost to follow up	15 (10.8)^d^
Outcomes	
Mortality	
Total	50 (36.0)^d^
Men	32 (64.0)^e^
Amputations	
Total of 1st amputations	71 (51.1)^d^
Minor	55 (77.5)^e^
Subsequent amputation	64 (90.1)^f^

^a^Percentage of the total of 3310 cultures

^b^Percentage of the total of 2238 positive cultures

^c^Percentage of the total number of 459 episodes

^d^Percentage of the total population of 139 people

^e^Percentage of the 50 deceased people

^f^Percentage of total of 71, 1st amputation

^g^Percentage of total of 71 participants with a 1st amputation

### Episodes, sampling method and outcomes

The second part of Table 2 shows the total number of episodes during the study and the median and interquartile range of these episodes per person. Of 155 episodes with osteomyelitis, the best sampling method was bone biopsy in 48 (31%); ulcer bed biopsy in 55 (35.5%) and swab in 52 (33.5%) of the episodes. Data regarding the presence of osteomyelitis were missing for 10 episodes (2.2%).

### Association between bacterial profiles and, amputation and mortality

Incidence rates and 95% confidence intervals for infection per profile per person year for profiles 1–5 were: 1.1 (0.99–1.2); 0.48 (0.41–0.57); 0.99 (0.88–1.1); 0.37 (0.31–0.45); 0.09 (0.06–0.13), respectively. HR’s of the time-dependent Cox regression for the association between bacterial profiles and, amputation and mortality including 95% CI’s are displayed in Table 3. Best sampling method appeared to be an effect modifier in the relation between infection with bacterial profile 2 and amputation risk; therefore, stratified results are presented for profile 2 (Table 3). In these stratified analyses we found for coagulase-negative *Staphylococcus*, *Cutibacterium*, *Corynebacterium, Enterococcus* (profile 2) in swabs a HR of 5.87 (2.9–11.8) for amputation. For gram-negative bacteria (profile 3), we found a HR of 2.6 (1.4–4.8) for mortality. Best sampling method did not appear an effect modifier in the association between bacterial profiles and mortality. Osteomyelitis was not an effect modifier in either association.

**Table 3:** Association between bacterial profiles and, amputation and mortality

	Amputation	Mortality
Profile	HR (95% CI)	HR (95% CI)
1	0.66 (0.39–1.1)	0.89 (0.49–1.6)
2	2.8 (1.7–4.6)	0.73 (0.38–1.4)
	Bone biopsy 0.84 (0.26–2.7)	
	Ulcer bed biopsy 0.89 (0.34–2.3)	
	Swab 5.9 (2.9–11.8)^a^	
3	1.3 (0.78–2.1)	2.6 (1.4–4.8)^B^
4	1.6 (0.91–2.6)	1.1 (0.58–2.2)
5	1.6 (0.58–4.5)	0.80 (0.19–3.3)

Table 3 shows Hazard Ratios (HR’s) and 95% Confidence Intervals (95% CI’s) for amputation and mortality according to bacterial profile. The association between profile 2 and amputation risk was modified by sampling method; therefore, separate HR’s for bone-, ulcer bed biopsy and swab are displayed. Profile 2 when present in swab

^a^Cultures were significantly associated with a higher amputation risk. ^B^Cultures were significantly associated with a higher mortality risk.

In the sensitivity analyses, we included 313 episodes in participants without history of amputation. These analyses showed similar associations between bacterial profiles and amputation or mortality risk as the primary analyses.

## Discussion

This study showed that in  people with DFI, there was no association between any of the bacterial species (profiles 1–5)  when found in ulcer bed or bone cultures as best sampling method, and risk of amputation. We did find an increased risk of amputation in people with DFI with coagulase-negative *Staphylococcus*, *Cutibacterium*, *Corynebacterium, Enterococcus* (profile 2), when best sampling method was swab. We also found that infection with gram-negative bacteria (profile 3) increased mortality risk. The method of sampling did not modify this association.

There was no association between any of the bacterial species (profiles 1–5) and risk of amputation when found in the sampling methods recommended by international guidelines, i.e., bone and ulcer bed biopsies. Only when these samples were not available, we looked at bacteria in swab cultures as best available sampling method. We found an association between culture of a swab sample with coagulase-negative *Staphylococcus*, *Cutibacterium*, *Corynebacterium, Enterococcus* (profile 2) and a higher amputation risk. This was an interesting observation, since these bacterial species, when found in swabs, are considered not pathogenic, colonizers or contaminants and are not always reported nor treated. Furthermore, these species are usually difficult to treat due to inherent or acquired antimicrobial resistance. However, when found in an aseptically obtained bone biopsy, these bacteria are considered pathogens and treated accordingly. Previous studies show that the concordance between swab and ulcer bed biopsy and bone biopsy is low [17, 20, 28]. Superficial cultures can miss pathogens causing infection deeper in the tissue and might identify colonizing agents as pathogens. Determining whether a cultured bacterium is a true pathogen or not, based on swab cultures is, therefore, difficult and will likely lead to over and under treatment and worse outcomes [30, 31]. This implicates, that the role of coagulase-negative *Staphylococcus*, *Cutibacterium*, *Corynebacterium,* and *Enterococcus* in DFI can be underestimated based on swab cultures, and stresses the importance of obtaining adequate samples, i.e., ulcer bed- and bone biopsies [5, 23–25, 32, 33]. Interpretation of these cultures is less ambiguous and will lead to better guided treatment [19]. Another possible explanation for a higher amputation risk for bacteria found in swabs is, that people with only swab cultures, were treated differently and probably not by our multidisciplinary foot care team, where ulcer bed biopsies and bone biopsies are common practice.

We found higher mortality rates in people with infection with gram-negative bacteria (profile 3), regardless of sampling method. The latter probably because gram-negative bacteria are always interpreted as pathogenic bacteria and treated accordingly. The association we found, is in line with the retrospective studies of Lipsky et al. and Shaheen et al [34, 35]. It is unclear whether gram-negative bacteria increase the risk of mortality directly or indirectly. The risk of mortality could be increased directly due to the pathogenicity of the gram negative bacteria, or indirectly, because people with an increased mortality risk due to comorbidities (e.g., peripheral artery diseases) are prone for infection with gram-negative bacteria.

*Staphylococcus aureus* and gram-negative bacteria are reported in a meta-analysis of Macdonald et al., as the main pathogen in DFI [36]. Our study confirmed these results, as profile 1 (consisting of *Staphylococcus aureus* and *Streptococcus)* and 3 (gram-negative bacteria), were most frequently cultured. In accordance with other studies we found that *Streptococcus and Staphylococcus aureus *(profile 1) do not increase amputation or mortality risk [7, 8, 34, 35]. Possible explanations are: the type of infection these bacteria cause, usually acute and mild, as a result of which infection will be recognised in an early stage and treatment starts promptly. *Streptococcus and Staphylococcus aureus* are always recognised as pathogens regardless of sampling method, these pathogens are covered by empirical treatment, and these bacteria are predominantly seen in people not recently treated with antibiotic therapy [5, 22, 37, 38]. Recent and past antibiotic use (long term or frequently), can influence culture results, e.g., due to bacterial selection, induction of antibiotic resistance or bacterial suppression [39, 40]. If bacterial growth is suppressed, and bacteria are not fully eradicated, this might lead to false negative culture results and inadequate treatment. To date there is limited research on the role of recent or past antibiotic use and the influence on culture results and infection remission in people with DFI.

It is difficult to investigate the association between a single bacterium and outcome. To be able to investigate this association, extremely large databases are needed to control for smalls groups (not all bacteria are cultured frequently), and multiple testing. To overcome these difficulties, we aggregated bacterial species into five profiles according to morphology and perceived virulence. The strength of this approach is, that we created 5 common profiles that are applicable for everyday practice, and results of the study are apprehensible. We also aggregated data into episodes of infection. Cultures taken from the same patients in 3 month time were considered as one episode. DFI’s usually occur in slow healing ulcers, by creating episodes we prevented that multiple cultures over time, of the same DFI, of one participant were in included in the analyses as separate infections. The weakness of this approach is, that it is somewhat artificial, and the cutoff, of 3 months as one infection episode, can be arbitrary in some cases of ongoing osteomyelitis or frequently relapsing infection.

Although infection increases the risk of amputation and amputation increases the risk of mortality, [12–15] we did not find an increased mortality risk in participants with profile 2 infections. Mortality depends on a combination of factors, and type of causative bacteria of DFI, is one of these factors. Other factors are, e.g., the presence of vascular disease and immobility [13]. Comorbidities might differ between participants with coagulase-negative *Staphylococcus*, *Cutibacterium*, *Corynebacterium, Enterococcus* (profile 2) and gram-negative infection (profile 3). Another explanation lies in the nature of the analyses. Participants with infection with one profile, could also have infection with other bacterial profiles, the combinations of these profiles vary between the separate analyses, and therefore, results cannot be combined.

In conclusion, in this retrospective cohort study in people with DFI, we found that there was no association between *Streptococcus* and *Staphylococcus aureus* (profile 1), coagulase-negative *Staphylococcus, Cutibacterium*, *Corynebacterium, Enterococcus* (profile 2), gram negative- (profile 3), anaerobe- (profile 4) and other gram positive bacteria (profile 5) and amputation when found in ulcer bed- or bone biopsies. Only when found in swab cultures, coagulase-negative *Staphylococcus, Cutibacterium*, *Corynebacterium, Enterococcus* (profile 2) were associated with a higher amputation risk. Infection with gram negative bacteria (profile 3) was associated with a higher mortality risk, regardless of sampling method. The results of this study propagate the importance of using a sampling method recommended by international guidelines to obtain cultures to optimize treatment and to lower the risks of amputation and mortality.

## Data Availability

All anonymised data is available upon reasonable request. For data requests, please contact the 1st author.
